# 血液肿瘤免疫及靶向药物治疗相关性感染预防及诊治中国专家共识（2025年版）

**DOI:** 10.3760/cma.j.cn121090-20241114-00451

**Published:** 2025-01

**Authors:** 中华医学会血液学分会感染学组 中华医学会血液学分会淋巴细胞疾病学组 中国临床肿瘤学会淋巴瘤专家委员会

## Abstract

随着新药不断问世，免疫治疗和分子靶向药物在血液系统恶性肿瘤治疗领域显现广泛的应用前景。本版专家共识依据最新国内外循证医学证据，再次组织专家们对感染的诊断、预防及治疗推荐意见进行更新，特别纳入了近期在国内上市或即将上市的抗体和小分子靶向药物，以及针对新型冠状病毒感染的药物管理意见。同时，本指南亦参照牛津大学循证医学中心的分级标准和推荐强度，对推荐意见的格式进行了相应调整。

## Abstract (en)

As novel therapeutic agents continue to emerge, immunotherapy and molecular-targeted drugs demonstrate expanding application prospects in hematological malignancy treatment. This expert consensus revision incorporates the latest evidence-based medicine from domestic and international sources, updating recommendations for infection diagnosis, prevention, and treatment. The document integrates recommendations for recently launched or imminent antibodies and small molecule targeted compounds, including COVID-19 considerations. This format of recommendations is modified according to the levels of evidence of The Oxford Centre for Evidence-Based Medicine (CEBM).

修订要点

·修改推荐意见格式，根据牛津大学循证医学中心的证据分级与推荐强度标准。

·新增近期国内上市或即将上市的抗体类及小分子靶向药物感染管理。

·新增新型冠状病毒感染下药物管理建议。

为规范和指导血液系统恶性肿瘤免疫及小分子靶向药物治疗相关感染的诊治，中华医学会血液学分会与中国临床肿瘤学会（CSCO）淋巴瘤专家委员会组织专家，基于美国国家综合癌症网络（NCCN）和欧洲白血病感染协作组（ECIL）针对免疫及靶向药物治疗相关感染管理的专家共识[1]–[2]，结合相关临床研究进展和国内情况制定了《血液肿瘤免疫及靶向药物治疗相关性感染预防及诊治中国专家共识（2021年版）》。近年来，免疫及靶向药物进展迅速，同时新型冠状病毒（以下简称SARS-CoV-2）全球范围内流行为血液恶性肿瘤患者带来了严重困扰。因此，根据国内外最新的循证医学证据再次组织专家对感染诊断、预防及治疗的推荐意见进行更新。

一、推荐等级

本共识采用2009版牛津大学循证医学中心的证据分级与推荐强度标准对每个临床问题的证据质量和推荐强度进行分级[3]，见表1。

**表1:** 2009版牛津大学证据级别与推荐意见强度的分级标准

推荐强度	证据级别	描述
A	1a	随机对照试验（RCT）的系统评价
	1b	结果可信区间小的RCT
	1c	显示“全或无”效应的任何证据
B	2a	队列研究的系统评价
	2b	单个队列研究（包括低质量的RCT，如失访率>20％）
	2c	基于患者结局的研究
	3a	病例对照研究的系统评价
	3b	单个病例对照研究
C	4	病例系列报告、低质量队列研究和低质量病例对照研究
D	5	专家意见（即无临床研究支持的仅依据基础研究或临床经验的推测）

二、抗体药物相关的感染

已经上市和即将在国内上市的抗体药物包括单克隆抗体、免疫检查点抑制剂、抗体偶联类药物（ADC）和双特异性抗体四大类。具体药物对免疫系统的影响及相关感染流行病学详见表2。

**表2:** 抗体药物相关感染

类别	免疫系统影响	代表药物	感染事件
抗CD20单抗	①B细胞减少相关低丙种球蛋白血症； ②中性粒细胞减少	利妥昔单抗、奥妥珠单抗	利妥昔单抗单药时，肺炎（4％）；联合CHOP方案时，≥3级感染（4.7％）[4]。 奥妥珠单抗单药时，肺炎（2.7％）[5]。 结核分枝杆菌、念珠菌、PcP、HBV再激活、CMV、VZV、JC病毒相关PML亦有报道
抗CD38单抗	①B细胞减少相关低丙种球蛋白血症； ②中性粒细胞减少	达雷妥尤单抗、伊沙妥昔单抗	达雷妥尤单抗单药主要引起<3级的上呼吸道感染（21％）[6]；与化疗药物和（或）靶向药物联合应用增加感染风险，≥3级感染（23％～30.7％）[7]，上呼吸道感染（2％～26％），肺炎（8％～15％），复发难治者感染风险更高。 伊沙妥昔单抗与靶向药物联合应用时，上呼吸道感染（7％～9％），肺炎（25％～26％）[8]–[9]。 VZV和HBV再激活风险高
抗CD19单抗	中性粒细胞减少	坦昔妥单抗	联合来那度胺，FN（12％），感染（73％，包括细菌、真菌和病毒）、≥3级肺炎（7％）[10]；针对中国人群感染发生率更高，≥3级肺炎（25％）
抗CD30 ADC	①影响T细胞亚群比例平衡； ②短暂的剂量依赖性中性粒细胞减少	维布妥昔单抗	常见感染为肺炎（10％）、HHV（1％～10％）、易出现HBV再激活、PcP（0.1％～1％）[11]、JC病毒相关PML可诱导死亡
抗CD79b ADC	中性粒细胞减少	维泊妥珠单抗	单药肺炎（4.4％）；联合BR方案，≥3级感染（23.1％～27.2％）；联合R-CHP方案，≥3级感染（14％），亚洲人群感染数据与整体人群类似[12]–[13]；严重感染包括败血症、肺炎（包括PcP和其他真菌性肺炎），HSV/VZV、CMV、HBV再激活和JC病毒感染导致的PML亦有报道
抗CD22 ADC	中性粒细胞减少	奥英妥珠单抗	单药≥3级FN（12％～53.3％，与疾病负担呈正比）、≥3级感染（22.8％～40％，严重感染包括克雷伯菌血症、大肠埃希菌败血症和感染性休克）[14]–[15]；联合化疗时，感染（17％～66％），真菌感染包括肺曲霉病、口腔念珠菌病亦有报道[16]
抗CD19 ADC	中性粒细胞减少	Loncastuximab tesirine	单药FN（3％），≥3级感染（10％），败血症和肺炎最常见，机会性感染亦有报道；联合利妥昔单抗时，严重感染（30％）[17]
抗CD33 ADC	中性粒细胞减少	吉妥珠单抗	单药FN（18％），感染（42％）；联合标准化疗，FN（24％～32％），感染（36％～55％），肺炎（25％）和败血症（29％）[18]
抗BCMA ADC	①B细胞减少； ②中性粒细胞减少症	Belantamab mafodotin	与免疫抑制剂和（或）靶向药物联合，≥3级感染（19.5％～49％），肺炎（4％），SARS-CoV-2（1％，肺炎<1％）[19]
PD-1/PD-L1单抗	药物没有增加感染的风险，但irAE需要合并使用类固醇类药物，导致潜伏性感染再激活[20]	纳武利尤单抗、帕博利珠单抗、特瑞普利单抗、信迪利单抗、替雷利珠单抗	合并使用免疫抑制药物致机会性感染（7.3％）、结核分枝杆菌、组织胞浆菌病、李斯特菌病[21]；治疗期间感染需与irAE鉴别，参考《中国临床肿瘤学会（CSCO）免疫检查点抑制剂相关的毒性管理指南》
CD19×CD3双抗	①B细胞减少相关低丙种球蛋白血症； ②中性粒细胞减少	贝林妥欧单抗	单药感染（17.8％～45％）[22]–[23]，真菌感染较为罕见，长期连续输注期间（2～4周）需关注静脉导管相关感染
CD20×CD3双抗	①B细胞减少相关低丙种球蛋白血症； ②中性粒细胞减少（持续≥30 d中性粒细胞减少率11.0％）	格菲妥单抗、莫妥珠单抗、艾可瑞妥单抗	格菲妥单抗FN（2.6％～3％），严重感染（15％～18.2％），以病毒性感染为主，SARS-CoV-2相关肺炎或SARS-CoV-2、VZV等，细菌性感染相对少见，IFD相对罕见[24]–[25]； 莫妥珠单抗相关感染事件包括尿路感染、败血症、肺炎、EB病毒和SARS-CoV-2感染； 艾可瑞妥单抗FN（2.5％），严重感染（15％），包括SARS-CoV-2（20％）、肺炎（13％）和机会性感染[26]
BCMA×CD3双抗	①B细胞减少相关低丙种球蛋白血症； ②中性粒细胞减少	特立妥单抗、埃纳妥单抗	特立妥单抗单药≥3级感染（22.4％），包括SARS-CoV-2（6％）、肺炎（15％），严重腺病毒和PcP感染[27]； 埃纳妥单抗单药≥3级感染（27.3％～39.8％），≥3级肺炎（3.3％）；机会性感染（9.1％），包括PcP、腺病毒感染、CMV再激活等；中断治疗的感染事件中，SARS-CoV-2感染最常见（25.2％）[28]
GPRC5D×CD3双抗	①B细胞减少相关低丙种球蛋白血症； ②中性粒细胞减少	Talquetamab	单药≥3级感染（15％～20％），致命感染（3.2％），包括SARS-CoV-2、败血症和真菌感染，而机会性感染发生率较低；联合达雷妥尤单抗，≥3级感染（27.5％～28.6％）[29]

**注** ADC：抗体偶联类药物；CHOP：环磷酰胺+阿霉素+长春新碱+泼尼松；BR：苯达莫司汀+利妥昔单抗；R-CHP：利妥昔单抗+环磷酰胺+阿霉素+泼尼松；PcP：肺孢子菌肺炎；HBV：乙型肝炎病毒；CMV：巨细胞病毒；HSV：单纯疱疹病毒；VZV：水痘-带状疱疹病毒；PML：进行性多灶性白质脑病；FN：中性粒细胞减少性发热；HHV：人类疱疹病毒；SARS-CoV-2：新型冠状病毒；IFD：侵袭性真菌病；irAE：免疫相关不良反应

三、小分子靶向药物相关性感染

常用小分子靶向药物包括酪氨酸激酶抑制剂（TKI）、蛋白酶体抑制剂（PI）、布鲁顿酪氨酸激酶（BTK）抑制剂、组蛋白去乙酰化酶（HDAC）抑制剂、JAK激酶抑制剂、BCL-2抑制剂、FLT3抑制剂、磷脂酰肌醇3激酶（PI3K）抑制剂、异柠檬酸脱氢酶（IDH-1/IDH-2）抑制剂、Smoothened蛋白（SMO）抑制剂、DNA甲基转移酶抑制剂和核输出蛋白1（XPO1）抑制剂等，其对免疫系统的影响及相关感染流行病学详见表3。

**表3:** 小分子靶向药物相关性感染

类别	免疫系统影响	代表药物	感染事件
TKI	①抑制非靶向激酶，造成CD4^+^和CD8^+^细胞增殖受抑制； ②抑制B细胞功能； ③中性粒细胞减少	伊马替尼、达沙替尼、尼洛替尼、奥雷巴替尼	伊马替尼相关感染（14％，主要发生在中性粒细胞减少期），肺炎（2％～4％），VZV（2.0％～7.0％）[30]–[31]； 尼洛替尼（Nilotinib）相关感染（7.9％）； 达沙替尼治疗相关感染风险最高（51％，其中肺炎和软组织感染最常见，治疗3个周期后风险逐渐上升），急性淋巴细胞白血病或接受大剂量皮质类固醇治疗时感染风险更大[32]； 奥雷巴替尼引起的严重感染事件中，肺炎（3.0％），上呼吸道感染（1％）[33]； TKI治疗相关机会性感染，包括EBV、结核分枝杆菌、诺卡菌病、PcP、人类细小病毒B19和CMV、HBV再激活
蛋白酶体抑制剂	①选择性耗竭T细胞； ②中性粒细胞减少	硼替佐米、伊沙佐米、卡非佐米	与免疫靶向药物联合应用致感染风险增加，肺炎（4％～18％）；VZV感染（6.0％～22.3％）；流感住院率66.7％，重症监护病房住院率为41.6％[1]；机会性感染如诺卡菌病、原藻病或PcP；HBV再激活
BTK抑制剂	①抑制B细胞发育，低丙种球蛋白血症； ②抑制Toll样受体介导的感染	伊布替尼、泽布替尼、奥布替尼、阿可替尼	伊布替尼≥3级感染（一线治疗13％～36％，复发/难治24％～51％，6个月内感染发生率最高），上呼吸道感染、泌尿道感染和鼻窦炎最常见，严重感染以肺炎最常见（25％）[34]–[35]；IFD、隐球菌感染、VZV再激活、PcP和EBV驱动的噬血细胞综合征均有报道； 根据关键临床研究数据，感染风险可随靶点特异性的增加而下降，泽布替尼、奥布替尼、阿可替尼治疗相关≥3级感染发生率分别为21.3％、15.4％和19％；但在国内真实世界回顾性研究数据中，伊布替尼、泽布替尼和奥布替尼单药或联合方案的总感染率分别为43.1％、34.0％和44.4％，三者的差异无统计学意义[36]； BTK抑制剂可造成HBV再激活，发生率为0.1％～8.1％
HDAC抑制剂	抑制Toll样受体介导的树状突细胞和巨噬细胞功能（传感、吞噬、细胞因子产生、黏附）	西达本胺	均见于联合用药，感染轻中度并可控
JAK激酶抑制剂	①抑制树突状细胞及CD4^+^ T细胞功能，降低Treg数量； ②抑制NK细胞	芦可替尼（JAK2抑制剂）；戈利昔替尼（JAK1抑制剂）	治疗MF时，芦可替尼≥3级感染（45％，包括细菌78％，病毒11％，真菌2％，脾肿大伴国际预后评分系统中危-2及以上的患者感染更重）[37]，VZV感染率随治疗时间延长而增加（1.9％上升至11.5％）；治疗GVHD时，病毒感染最为常见（36.0％），其中CMV（18.7％），EBV（10.8％），可见HBV再激活，细菌感染（5.5％），真菌感染（3.4％）[38]。 戈利昔替尼≥3级感染（9％），肺炎最常见（7％），病毒感染以VZV最为常见（5％）。 考虑PcP感染风险，常规建议患者预防[39]
BCL-2抑制剂	中性粒细胞减少	维奈克拉	≥3级感染（淋巴瘤治疗：17.7％~19.0％；髓系肿瘤治疗：72％~74％，≥3级IFD 8％）；中国患者感染风险略低，≥3级感染（43.3％～67.0％），肺炎（22％～23.3％）[40]–[41]
FLT3抑制剂	①中性粒细胞减少； ②抑制树状突细胞功能，特别是异基因造血干细胞移植患者中	吉瑞替尼、奎扎替尼、米哚妥林、索拉非尼	吉瑞替尼单药≥3级FN（40.0％～46.7％），肺炎（4.0％～14.4％），严重感染（1.2％），≥3级真菌感染（4％）；与化疗药物和（或）靶向药物（维奈克拉）联合[42]–[43]，≥3级FN（48.7％～63.3％），严重肺炎（13.9％），IFD（25％）。 奎扎替尼单药≥3级FN（7％），肺炎（1％~12％），IFD（3％）；联合化疗时感染风险增加，≥3级FN（41％~47％），肺炎（12％~21％，严重肺炎4％），其他细菌感染包括败血症、艰难梭菌结肠炎、尿路感染、蜂窝织炎、感染性休克等。 米哚妥林FN（20％～82％），肺炎最常见（2％～23％），细菌感染多见于联合化疗时，真菌和病毒（HHV）感染少见。 索拉非尼单药维持不增加EBV和CMV感染风险；联合强化疗，≥3级FN（65％），≥3级感染（55％）[44]–[45]
PI3K抑制剂	①作用于T细胞和NK细胞，易引起免疫抑制； ②中性粒细胞减少	林普利塞	严重肺炎（12％），发生的中位时间为5（1～14）个月；PcP（0.6％）；CMV、HBV、VZV均有被激活的风险[46]
IDH抑制剂	无特异性免疫抑制相关报告	艾伏尼布、恩西地平	单药治疗AML不增加感染风险；联合强化疗时，艾伏尼布≥3级感染（5.0％~5.7％），恩西地平≥3级感染（6.5％），但药物与感染事件的相关性尚无定论，且治疗期间出现发热、急性呼吸窘迫或肺浸润等症状时，首先需与严重的分化综合征相鉴别[47]
SMO抑制剂	无特异性免疫抑制相关报告	格拉吉布	单药不增加感染风险；联合小剂量阿糖胞苷，≥3级FN（35.7％），肺炎（28.6％）[48]；联合强化疗，≥3级FN（53.5％），肺炎（20.4％）[49]
DNA甲基转移酶抑制剂	中性粒细胞减少	地西他滨、阿扎胞苷	单药地西他滨，肺炎（20.60％）；单药阿扎胞苷，肺炎（6.40％）[50]；阿扎胞苷联合维奈克拉治疗老年AML，≥3级FN（43％），肺炎（26％），败血症（7％）[51]
XPO1抑制剂	治疗7 d内开始出现中性粒细胞减少，但感染与中性粒细胞减少的相关性需要进一步研究	塞利尼索	FN（1％~3％）[52]；机会性感染包括但不限于IFD和病毒

**注** TKI：酪氨酸激酶抑制剂；BTK：布鲁顿酪氨酸激酶；HDAC：组蛋白去乙酰化酶；PI3K：磷脂酰肌醇3激酶；IDH：异柠檬酸脱氢酶；SMO：Smoothened蛋白；XPO1：核输出蛋白1；Treg：调节性T细胞；PcP：肺孢子菌肺炎；CMV：巨细胞病毒；HBV：乙型肝炎病毒；VZV：水痘-带状疱疹病毒；EBV：EB病毒；FN：中性粒细胞减少性发热；HHV：人类疱疹病毒；IFD：侵袭性真菌病；MF：原发性骨髓纤维化；GVHD：移植物抗宿主病；AML：急性髓系白血病

四、感染筛查与诊断

（一）治疗前感染筛查

根据不同抗体和小分子靶向药物相关感染流行病学特征进行治疗前感染筛查（筛查重点见表2和表3）：①乙型肝炎病毒（HBV）、丙型肝炎病毒、梅毒、人类免疫缺陷病毒；②人类疱疹病毒（HHV）1/2、HHV-3（水痘-带状疱疹病毒，VZV）、HHV-4（EB病毒，EBV）、HHV-5（巨细胞病毒，CMV）、JC病毒；③红细胞沉降率；④结核分枝杆菌（结核特异性细胞免疫三项、TB SPOT、PPD试验等）；⑤呼吸道相关病毒（有上呼吸道症状者）；⑥半乳甘露聚糖抗原试验/1,3-β-D-葡聚糖试验（GM/G试验）。

（二）感染的诊断

建议参考《中国中性粒细胞缺乏伴发热患者抗菌药物临床应用指南（2020年版）》[53]，注意与免疫相关不良反应（irAE）及严重的分化综合征鉴别。

1. 病史询问和体格检查：详细了解既往靶向和免疫治疗药物情况、抗生素使用和定植情况，发现感染的高危和隐匿部位。

2. 实验室检查：全血细胞计数、肝肾功能和电解质检查、免疫球蛋白；降钙素原、C反应蛋白、IL-6等感染相关指标的检查对诊断有提示意义。

3. 病原学检查：

（1）血培养［至少同时行两份血培养检查，如果存在中心静脉导管（CVC），一份血标本从CVC的管腔采集，另一份从外周静脉采集。无CVC者，应采集不同部位静脉的两份血标本进行培养，采血量为每瓶10 ml。如果经验性抗菌药物治疗后患者仍持续发热，可以每2～3 d进行1次重复培养］。

（2）痰培养、粪培养、尿培养、脑脊液培养（必要时）。

（3）HBV、HHV、JC病毒、SARS-CoV-2。

（4）呼吸道相关病毒（有上呼吸道症状者）。

（5）GM/G试验。

（6）结核分枝杆菌（结核特异性细胞免疫三项、TB SPOT、PPD试验等）。

（7）支气管镜检查，肺泡灌洗。

（8）聚合酶链反应（PCR）、宏基因组二代测序（mNGS）、TORCH。

4. 影像学检查：

（1）胸部高分辨CT；

（2）头颅MRI；

（3）腹部B超或CT。

五、预防与治疗

感染预防的总体原则见图1。

**图1:**
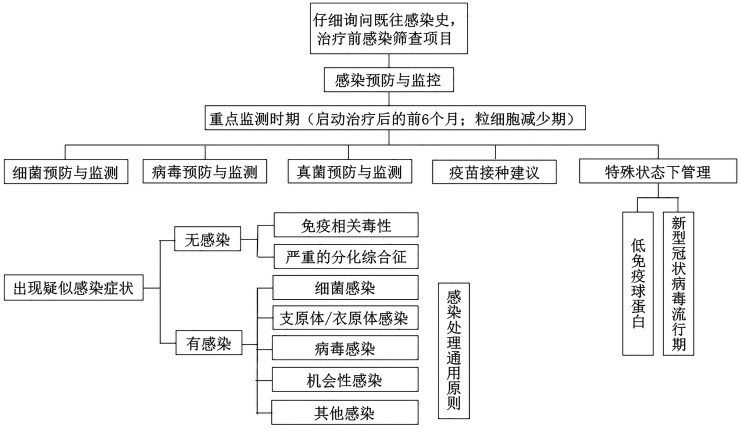
感染预防总体原则

（一）感染预防与监测

推荐意见1：部分感染事件主要发生于启动治疗后前6个月，粒细胞减少期，需重点监测（推荐强度B，证据级别2a）。

1. 细菌预防与监测：目前尚无循证医学证据支持在靶向治疗的同时予常规细菌预防。启动靶向治疗后，根据临床情况，定期监测血常规、免疫球蛋白及降钙素原、C反应蛋白、IL-6等感染相关指标。

2. 病毒预防与监测：根据相关临床数据、国内外指南和药物说明，对不同类型免疫治疗和靶向药物治疗后HBV再激活[54]–[55]、VZV、CMV、EBV及JC病毒感染风险提出预防及监测建议，具体见表4。

**表4:** 病毒预防与监测建议

类别	HBV	VZV	CMV	EBV	JC病毒
抗体药物相关病毒感染					
抗CD20单抗	推荐预防	推荐预防	推荐监测	-	警惕PML提示症状，如果确诊，则应永久终止治疗
抗CD38单抗	推荐预防	推荐预防	-	-	-
抗CD19单抗	推荐预防	推荐预防	推荐监测	-	-
抗CD30 ADC	推荐预防	推荐预防	推荐监测	-	警惕PML提示症状，如果确诊，则应永久终止治疗
抗CD79b ADC	推荐预防	推荐监测	推荐监测	-	警惕PML提示症状，如果确诊，则应永久终止治疗
抗CD22 ADC	推荐监测	-	-	-	-
抗CD19 ADC	推荐预防	推荐监测	推荐监测	-	-
抗CD33 ADC	推荐监测	-	-	-	-
抗BCMA ADC	推荐预防	推荐预防	推荐监测	-	-
PD-1/PD-L1单抗	HBV DNA>100 IU/ml，推荐预防	推荐监测	推荐监测	-	-
CD19×CD3双抗	推荐预防	推荐监测	推荐监测	推荐监测	-
CD20×CD3双抗	推荐预防	推荐预防	推荐监测	推荐监测	-
BCMA×CD3双抗	推荐预防	推荐预防	推荐监测	推荐监测	-
GPRC5D×CD3双抗	推荐预防	推荐预防	推荐监测	推荐监测	-
分子靶向药物相关病毒感染					
TKI	推荐预防	推荐预防	推荐监测	推荐监测	-
蛋白酶体抑制剂	推荐预防	推荐预防			-
BTK抑制剂	推荐预防	推荐预防	推荐监测	推荐监测	警惕PML提示症状，如果确诊，则应永久终止治疗
HDAC抑制剂	推荐预防	-	-	-	-
JAK激酶抑制剂	推荐预防	推荐预防	推荐监测	推荐监测	警惕PML提示症状，如果确诊，则应永久终止治疗
BCL-2抑制剂	推荐监测	-	-	-	-
FLT3抑制剂	推荐监测	-	-	-	-
PI3K抑制剂	推荐预防	推荐预防	推荐监测	-	-
IDH抑制剂	推荐监测	-	-	-	-
SMO抑制剂	推荐监测	-	-	-	-
DNA甲基转移酶抑制剂	推荐监测	-	-	-	-
XPO1抑制剂	推荐预防	-	-	-	-

**注** ADC：抗体偶联类药物；TKI：酪氨酸激酶抑制剂；BTK：布鲁顿酪氨酸激酶；HDAC：组蛋白去乙酰化酶；PI3K：磷脂酰肌醇3激酶；IDH：异柠檬酸脱氢酶；SMO：Smoothened蛋白；XPO：核输出蛋白；HBV：乙型肝炎病毒；VZV：水痘-带状疱疹病毒；CMV：巨细胞病毒；EBV：EB病毒；PML：进行性多灶性白质脑病；-：无数据

推荐意见2：

①HBV预防：建议乙型肝炎表面抗原（HBsAg）阳性和（或）乙型肝炎病毒核心抗体（抗-HBc）阳性患者，最迟应在治疗前1周开始服用抗病毒药物（如恩替卡韦0.5 mg每日1次、丙酚替诺福韦25 mg每日1次或富马酸替诺福韦酯300 mg每日1次）（推荐强度A，证据级别1a）；疗程建议不少于12个月，至少应达到免疫及靶向治疗结束后6个月（推荐强度B，证据级别2b）。

②单纯疱疹病毒（HSV）/VZV预防：血清学检测IgG阳性者，在开始治疗至停药后至少4周建议予预防性抗病毒药物（如阿昔洛韦400 mg每日2次或伐昔洛韦300 mg每日2次），特别是粒细胞减少期（推荐强度B，证据级别2b）。

③CMV、EBV监测：血清学检测IgG阳性，IgM阴性者，治疗期间应每1～3个月进行病毒PCR监测；CMV预防推荐使用来特莫韦片480 mg每日1次（推荐强度C，证据级别4）。

④进行性多灶性白质脑病（PML）推荐的评估包括：警惕PML提示症状（如认知、神经或精神症状）。神经科会诊、钆增强脑磁共振成像和脑脊液JC病毒DNA的聚合酶链反应分析或证实存在JC病毒后行脑活检。若无其他替代诊断，应保证随访和评估。一旦确诊PML，应永久停药（推荐强度B，证据级别2c）。

3. 真菌预防与监测：根据相关临床数据、国内外指南和药物说明，对不同类型免疫治疗和靶向药物治疗相关的侵袭性真菌病（IFD）及非典型真菌感染（肺孢子菌肺炎，PcP）提出预防及监测建议，具体见表5。

**表5:** 真菌预防与监测建议

类别	侵袭性真菌病	肺孢子菌肺炎
抗体药物		
抗CD20单抗	推荐监测	推荐监测
抗CD38单抗	推荐监测	-
抗CD19单抗	推荐监测	-
抗CD30 ADC	推荐监测	推荐监测，根据当地标准预防
抗CD79b ADC	推荐监测	推荐监测，根据当地标准预防
抗CD22 ADC	推荐监测，特别警惕毛霉菌、曲霉菌	-
抗CD19 ADC	推荐监测	-
抗CD33 ADC	推荐监测	-
抗BCMA ADC	推荐监测	-
PD-1/PD-L1单抗	若糖皮质激素（泼尼松≥20 mg/d）应用≥6周，应考虑抗真菌预防	若合并糖皮质激素（泼尼松≥20 mg/d）应用≥4周，应考虑预防肺孢子菌肺炎治疗
CD19×CD3双抗	真菌感染罕见	-
CD20×CD3双抗	侵袭性真菌罕见	推荐监测，根据当地标准预防
BCMA×CD3双抗	粒细胞缺乏期建议预防	推荐监测，根据当地标准预防
GPRC5D×CD3双抗	粒细胞缺乏期建议预防	-
小分子靶向药物		
TKI	真菌感染罕见	推荐监测，但不常规预防
蛋白酶体抑制剂	真菌感染罕见	推荐监测，但不常规预防
BTK抑制剂	推荐监测，特别是中枢神经系统感染	推荐监测，但不常规预防
HDAC抑制剂	-	-
JAK激酶抑制剂	推荐监测	常规预防
BCL-2抑制剂	推荐检测，粒细胞缺乏期建议预防	-
FLT3抑制剂	推荐监测	-
PI3K抑制剂	推荐监测	治疗期间及停药后2～6个月常规预防
IDH抑制剂	-	-
SMO抑制剂	-	-
DNA甲基转移酶抑制剂	推荐检测，粒细胞缺乏期建议预防	推荐监测，根据当地标准预防
XPO1抑制剂	推荐监测	-

**注** ADC：抗体偶联类药物；TKI：酪氨酸激酶抑制剂；BTK：布鲁顿酪氨酸激酶；HDAC：组蛋白去乙酰化酶；PI3K：磷脂酰肌醇3激酶；IDH：异柠檬酸脱氢酶；SMO：Smoothened蛋白；XPO：核输出蛋白；-：无数据

推荐意见3：

①初级真菌预防推荐药物为氟康唑400 mg/d或泊沙康唑200 mg口服每日3次；PcP预防推荐药物为复方磺胺甲噁唑800 mg每日2次，每周2次（推荐强度A，证据级别1b）；预防时机推荐在抗肿瘤治疗期间及停药后2～6个月（推荐强度C，证据级别4）。

②irAE发生后通常需要使用免疫抑制剂，若糖皮质激素（泼尼松≥20 mg/d）应用≥4周，应考虑PcP预防；若糖皮质激素（泼尼松≥20 mg/d）应用≥6周，应考虑真菌预防（具体参考《NCCN免疫治疗相关毒性的管理指南》及《CSCO免疫检查点抑制剂相关的毒性管理指南》）（推荐强度B，证据级别2a）。

4. 疫苗接种：国际文献建议应尽可能在开始抗肿瘤治疗之前给患者接种疫苗，包括水痘/带状疱疹、流感和肺炎疫苗等。应具体评估患者潜在的风险和收益后做出选择，肿瘤活动期通常不建议接种疫苗，特殊疫苗接种按国家相关规定进行。

推荐意见4：

①血液系统恶性肿瘤患者应在免疫抑制治疗前至少4周接种疫苗，一般不建议接种减毒活疫苗，可以接种灭活疫苗（应答率可能下降）（推荐强度D，证据级别5）。

②新型冠状病毒疫苗接种按照《成人血液病患者新型冠状病毒疫苗接种中国专家共识（2023年版）》[56]指导。优先选择灭活疫苗，亦可考虑使用重组亚单位疫苗，一般禁止使用减毒活病毒载体疫苗（推荐强度B，证据级别2a）。

5. 特殊感染监测：包括结核分枝杆菌、组织胞浆菌病、李斯特菌病和诺卡菌病等；注意病史询问，特别是在常规抗感染药物治疗无效时，需考虑此类特殊病原菌感染可能。

6. 治疗期间特殊状态下感染管理建议：

（1）低丙种球蛋白血症状态管理

推荐意见5：对于IgG≤4 g/L且（或）合并严重或反复感染者，推荐每月重复1次静脉注射丙种球蛋白，并将IgG维持在4 g/L以上（推荐强度B，证据级别2b）。

（2）SARS-CoV-2流行期间：SARS-CoV-2感染会增加疾病进展和死亡的风险，特别是B细胞损伤或接受B细胞清除治疗的血液肿瘤患者，确定SARS-CoV-2感染后，一般应暂停并推迟抗肿瘤治疗（见表6），具体可参考《NCCN癌症相关感染指南》及《SARS-CoV-2感染的血液系统恶性肿瘤患者管理中国专家共识》[57]。

**表6:** 新型冠状病毒感染管理

患者类型	感染程度	暂停时间（自首次阳性检测结果日期开始）	重启指征	
			主要条件	次要条件
非紧急治疗需求，计划接受靶向治疗、免疫治疗的患者	无症状	暂停10 d	持续无症状	连续两次实时聚合酶链反应检测阴性（两次检测至少间隔24 h）
	轻-中度	暂停至少14 d	症状好转，并且在不使用退烧药的情况下退热至少24 h	
	重症-危重症	暂停至少21～28 d	症状好转，并且在不使用退烧药的情况下退热至少72 h	
需紧急启动治疗的患者			根据血液病专家判断进行治疗
可治愈的新诊断或复发侵袭性患者			根据指南对患者进行治疗，不建议延迟治疗起始时间
惰性或不可治愈的患者			根据肿瘤负荷进行观察随访，或选择免疫抑制较弱及住院需求少的方案

推荐意见6：

①SARS-CoV-2标准检测应采用实时聚合酶链反应（RT-PCR），只有在无法进行RT-PCR检测时才允许进行抗原检测（推荐强度B，证据级别2a）。

②常见的预防策略，包括（但不限于）手卫生、保持安全距离（1～2 m）和正确佩戴口罩（推荐强度A，证据级别1c）。

③确定SARS-CoV-2感染后，一般应暂停并推迟抗肿瘤治疗，根据临床SARS-CoV-2感染严重程度、血液肿瘤的类型和状态决定重启指征（推荐强度B，证据级别2c）；推荐连续两次RT-PCR检测阴性（两次检测至少间隔24 h）后再启动治疗（推荐强度C，证据级别4）。

④若由于肿瘤无法控制而迫切需要进行抗肿瘤治疗，则应根据临床医师的判断进行治疗。在抗肿瘤治疗过程中，应动态监测病毒RNA，谨防病毒再激活（推荐强度B，证据级别2c）。

（二）感染治疗原则

感染治疗原则见图2。

**图2:**
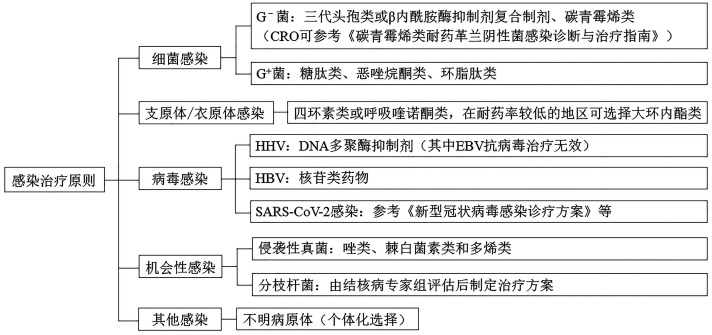
感染治疗原则 **注** 注意药物相互作用，特别是CYP3A4拮抗；G^-^菌：革兰氏阴性菌；G^+^菌：革兰氏阳性菌；CRO：碳青霉烯类耐药革兰氏阴性杆菌；HHV：人类疱疹病毒；EBV：EB病毒；HBV：乙型肝炎病毒；SARS-CoV-2：新型冠状病毒

1. 感染类型：

（1）细菌感染：①革兰氏阳性菌（G^+^菌）；②革兰氏阴性菌（G^-^菌）。

（2）衣原体/支原体。

（3）病毒感染。

（4）机会性感染：①IFD；②分枝杆菌。

（5）其他：不明病原感染。

2. 感染处理通用原则：

（1）细菌感染：具体可参考《中国中性粒细胞缺乏伴发热患者抗菌药物临床应用指南（2020年版）》[53]调整药物：①G^-^菌：三代头孢类或β内酰胺酶抑制剂复合制剂（如头孢曲松、头孢哌酮/舒巴坦、哌拉西林/他唑巴坦等），碳青霉烯类（如美罗培南、亚胺培南等）；碳青霉烯类耐药革兰氏阴性杆菌（CRO）感染管理可参考《碳青霉烯类耐药革兰阴性菌感染诊断与治疗指南》[58]；②G^+^菌：糖肽类（如万古霉素、替考拉宁）、恶唑烷酮类（利奈唑胺）、环脂肽类（达托霉素）等。

（2）支原体/衣原体感染：根据《中国成人社区获得性肺炎诊断和治疗指南（2016版）》[59]推荐管理，注意药物相互作用。

（3）病毒感染：①HHV（包括VZV、CMV）：DNA多聚酶抑制剂（如阿昔洛韦、伐昔洛韦、更昔洛韦、泛昔洛韦、膦甲酸钠和来特莫韦等）；EBV：尚无统一治疗方案，抗病毒治疗无效，可尝试免疫抑制治疗、细胞毒性药物化疗或细胞毒性T淋巴细胞治疗。②HBV再激活：核苷类药物（如恩替卡韦、丙酚替诺福韦、富马酸替诺福韦酯等）。③SARS-CoV-2感染：阿兹夫定、奈玛特韦/利托那韦和莫诺拉韦，注意药物相互作用［具体方案建议参考《新型冠状病毒感染诊疗方案（试行第十版）》及《SARS-CoV-2感染的血液系统恶性肿瘤患者管理中国专家共识》[57]］。

（4）机会性感染：①真菌感染，具体可参考《血液病/恶性肿瘤患者侵袭性真菌病的诊断标准与治疗原则（第六次修订版）》[60]：唑类（如伏立康唑、泊沙康唑和艾沙康唑等，注意药物相互作用）、棘白菌素类（卡泊芬净）和多烯类（两性霉素B脂质体）；②结核分枝杆菌：异烟肼、利福平、乙胺丁醇和吡嗪酰胺等，注意药物相互作用；建议由结核病专家组做详细的风险评估，根据传染源的耐药谱制定治疗方案。

（5）严密监测患者感染的情况，并及时对药物剂量作出调整。部分免疫治疗和靶向药与强效CYP3A4抑制剂有明显相互作用（表7），需暂时停药或者在密切监测下减量使用。

**表7:** 药物相互作用

类别	特殊药物合并使用时剂量调整建议	
	强效CYP3A4抑制剂^a^	强效CYP3A4诱导剂^b^
抗体药物		
抗CD20单抗	无	无
抗CD38单抗	无	无
抗CD19单抗	无	无
抗CD30 ADC	尚不明确	降低药物疗效
抗CD79b ADC	增加未结合的MMAE AUC 45％	降低未结合的MMAE AUC 63％
抗CD22 ADC	无	无
抗CD19 ADC	尚不明确	尚不明确
抗CD33 ADC	无	无
抗BCMA ADC	无	无
PD-1/PD-L1单抗	无	无
CD19×CD3双抗	无	无
CD20×CD3双抗	无	无
BCMA×CD3双抗	无	无
GPRC5D×CD3双抗	无	无
小分子靶向药物		
TKI	需减少给药剂量，监测用药反应	减低药物疗效，应避免同时使用
蛋白酶体抑制剂	无	无
BTK抑制剂	需减少给药剂量，监测用药反应	减低药物疗效，应避免同时使用
HDAC抑制剂	无	无
JAK激酶抑制剂	减少给药剂量约50％	减低药物疗效，应避免同时使用
BCL-2抑制剂	使维奈克拉血清水平增加约8倍	减低药物疗效，应避免同时使用
FLT3抑制剂	需减少给药剂量，密切监测QT间期延长	减低药物疗效，应避免同时使用
PI3K抑制剂	少部分通过CYP3A4酶代谢，监测用药反应	对体内暴露量影响有限
IDH抑制剂	替代或减少给药剂量，密切监测QTc间期延长	减低药物疗效，应避免同时使用
SMO抑制剂	无	无
DNA甲基转移酶抑制剂	无	无
XPO1抑制剂	无	无

**注** TKI：酪氨酸激酶抑制剂；BTK：布鲁顿酪氨酸激酶；HDAC：组蛋白去乙酰化酶；PI3K：磷脂酰肌醇3激酶；IDH：异柠檬酸脱氢酶；SMO：Smoothened蛋白；XPO：核输出蛋白；MMAE：一甲基澳瑞他汀E；AUC：曲线下面积。^a^ 唑类抗真菌药物—强效CYP3A4抑制剂：酮康唑、伊曲康唑、泊沙康唑、伏立康唑；中效CYP3A4抑制剂：氟康唑；CYP3A4弱抑制剂：艾沙康唑。大环内酯类抗生素—强效CYP3A4抑制剂：克拉霉素、泰利霉素、氯霉素；中效CYP3A4抑制剂：红霉素。其他中效CYP3A4抑制剂：异烟肼。新型冠状病毒药物—强效CYP3A4抑制剂：奈玛特韦/利托那韦。^b^ CYP3A4诱导剂：利福平、利福霉素、利福喷丁、利福昔明

推荐意见7：

①考虑到免疫治疗及靶向药物治疗患者存在免疫功能抑制，故感染后应尽快启动抗菌药物初始经验治疗，而不必等微生物学的结果（推荐强度B，证据级别2a）。

②支原体、衣原体感染患者建议口服多西环素或米诺环素（推荐强度B，证据级别3b）；我国肺炎支原体对大环内酯类药物耐药率高，在耐药率较低地区可用于经验性抗感染治疗；呼吸喹诺酮类可用于上述药物耐药率较高地区或药物过敏或不耐受患者的替代治疗（推荐强度B，证据级别2c）。

③HBV再激活：预防采用恩替卡韦者换用丙酚替诺福韦或富马酸替诺福韦酯，预防采用丙酚替诺福韦或富马酸替诺福韦酯者换用恩替卡韦，或2种药物联合使用（推荐强度A，证据级别1b）。

④建议在SARS-CoV-2症状出现后7 d内开始治疗（推荐强度B，证据级别2c）；病毒拷贝数居高不下者，可采取长期持续的抗病毒治疗和重复给药（推荐强度C，证据级别4）。

⑤不推荐维奈克拉联合唑类抗真菌药，有条件的中心建议在监测血药浓度的情况下调整剂量；联合伏立康唑/伊曲康唑/泊沙康唑用药时，维奈托克剂量至少减少75％（减至70～100 mg/d）；联合艾沙康唑/氟康唑时，维奈克拉剂量至少减少50％（推荐强度B，证据级别2b）。

⑥使用强效CYP3A4抑制剂时，建议暂停使用伊布替尼/泽布替尼/阿可替尼，或在严密监视血药浓度的情况下调整剂量（推荐强度B，证据级别2c）。

⑦与强效CYP3A4抑制剂同时用药时，芦可替尼每日总剂量应减少约50％，并严密监视血药浓度（推荐强度C，证据级别4）。

⑧奥英妥珠单抗、吉妥珠单抗与已知可延长QT间期或诱发尖端扭转型室性心动过速的药物合并使用时，可能增加QTc间期延长的心脏风险，需要临床警惕（推荐强度C，证据级别4）。
